# Incidence rates of cardiovascular outcomes in a community‐based population of cancer patients

**DOI:** 10.1002/cam4.2657

**Published:** 2019-10-30

**Authors:** Rajeev Masson, Lina Titievsky, Douglas A. Corley, Wei Zhao, Alfredo R. Lopez, Jennifer Schneider, Jonathan G. Zaroff

**Affiliations:** ^1^ Kaiser Permanente San Francisco Medical Center San Francisco CA USA; ^2^ Department of Epidemiology Pfizer Inc. New York NY USA; ^3^ Division of Research Kaiser Permanente Northern California Oakland CA USA

**Keywords:** cancer, heart failure, hypertension, myocardial infarction, venous thromboembolism

## Abstract

**Background:**

There are limited data on the incidence of cardiovascular disease among cancer patients in the pre‐tyrosine kinase inhibitor (TKI) era. Such data are important in order to contextualize the incidence of various cardiovascular outcomes among cancer patients enrolled in clinical trials of new agents and for postmarketing surveillance.

**Methods:**

A retrospective cohort study was conducted using data from the Kaiser Permanente Northern California (KPNC) population of cancer patients. The inclusion criterion was a KPNC Cancer Registry diagnosis of any of several selected solid and hematologic tumors between 1997 and 2009 not treated with a TKI. Endpoints were identified using ICD‐9 codes and included acute coronary syndrome, heart failure, stroke, cardiac arrest, hypertension, venous thromboembolism, all‐cause mortality, and cardiovascular mortality. Event rates were calculated according to type of cancer and number of cardiovascular risk factors.

**Results:**

The study included almost 165 000 individuals with a broad variety of tumor types. The parent cohort was 54% female and 35% were ≥70 years old. Cardiovascular risk factors such as diabetes mellitus (14% of patients with solid tumors, 15% of patients with liquid tumors), dyslipidemia (33%, 31%), hypertension (50%, 49%), and smoking (35%, 32%) were common. The most frequent adverse outcomes were incident hypertension (26.8‐61.0 cases per 1000 person‐years, depending on the type of cancer), heart failure (9.4‐78.7), and acute coronary syndrome (2.6‐48.1). These event rates are high compared to what has been reported in prior KPNC cohort studies of patients without cancer. The rates of acute coronary syndrome, heart failure, and ischemic stroke increased with increasing numbers of cardiovascular risk factors.

**Conclusions:**

In a population of patients with cancer not exposed to TKIs, cardiovascular risk factors and outcomes are very common, regardless of cancer type. These data can inform the evaluation of potential excess cardiovascular risks from new interventions.

## Graphical abstract

In a cancer population not exposed to tyrosine kinase inhibitors or bevacizumab, cardiovascular outcomes are common, regardless of cancer type. Cardiovascular events occur more frequently in cancer patients with increasing numbers of cardiovascular risk factors.

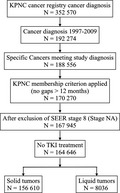

## 1 INTRODUCTION

With the advent of new chemotherapy agents, cancer patients are surviving longer but experiencing increased morbidity and mortality from cardiovascular disease. The number of cancer survivors in the United States has grown to over 10 million^1^ and there are limited data on the prevalence and incidence of cardiovascular disease in this population.^2^, ^3^, ^4^, ^5^, ^6^, ^7^ Chemotherapy agents, including increasingly popularized tyrosine kinase inhibitors (TKIs), may have cardiotoxic effects^8^, ^9^ but limitations of clinical trials (sample size, follow‐up time) and post‐marketing surveillance studies have made it difficult to determine to what extent the newer agents increase cardiovascular risk above the background level. The objective of this study was to quantify the incidence rates of cardiovascular outcomes among patients diagnosed with different types of cancer in the pre‐TKI and bevacizumab treatment era.

## 2 METHODS

### 2.1 Data sources

Kaiser Permanente Northern California (KPNC) is an integrated health program that included 3.2 million members at the time of this analysis. KPNC provides comprehensive care to its members, has high member retention rates (more than 80% at 10 years postcancer diagnosis), and is thus uniquely positioned to study cancer treatment, outcomes, and survivorship. The membership's demographics closely resemble the underlying census population of Northern California.^10^ Data were combined from a variety of KPNC research databases, including a cancer registry which includes specific cancer diagnoses, stages, and treatments.

### 2.2 Study population

**Figure 1:**
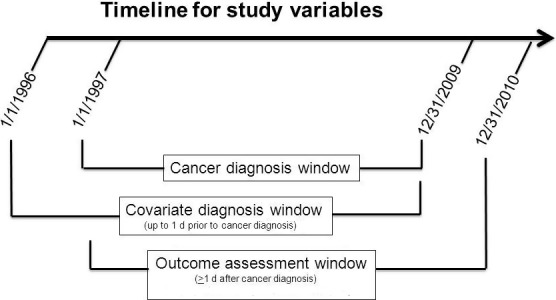
Timeline for study variables

For this study, patients were identified with selected solid tumor (excluding non‐melanoma skin cancer) and liquid tumor cancer diagnoses between 1997 and 2009 from the cancer registry. Patients with gaps in membership greater than 12 months, unknown cancer stage, or treatment with either a TKI or bevacizumab were excluded from the analysis. The timeline used for cohort inclusion, covariates, and outcomes is shown in Figure 1.

### 2.3 Risk factors for cardiovascular disease

Cardiovascular risk factors of interest included: hypertension (ICD‐9 codes 401.0, 401.1, 401.9), hyperlipidemia (ICD‐9 codes 272.0, 272.1, 272.2, 272.4), diabetes mellitus (ICD‐9 codes 250.0‐250.9, 250.0‐250.3), and a history of coronary artery disease (ICD‐9 code 414). The code of interest was required to be present between 1/1/1996 and the day before the cancer diagnosis (see Appendix Table S1 for coding details).

### 2.4 Demographic and clinical characteristics

Demographic factors and clinical characteristics such as smoking status, history of heart failure, and types of cancer treatment received were obtained from KPNC research databases (see Appendix Table S1 for coding details).

Primary endpoints were required to occur after the cancer diagnosis and included the following: Acute coronary syndrome (ACS, ICD9 codes 411.1 or 410), heart failure (428, 402.01, 402.11, 402.91), stroke (ischemic, 433.1, 434.1, 436 and hemorrhagic, 430‐432), cardiac arrest or sustained ventricular arrhythmia (427.1, 427.4‐5), hypertension (401.0‐1, 401.9), venous thromboembolism (deep venous thrombosis, 451.11, 451.19, 451.2, 451.40‐42, 451.81, 451.83, 451.89, 453.4 and pulmonary embolism, 415.1), all‐cause mortality, and CV‐specific mortality (ie death with one of the CV endpoints listed above as the primary cause or ICD10 death codes I00‐I99). Please see Appendix Table S1 for the detailed endpoint coding methodology.

Incidence rates of cardiovascular outcomes were also stratified by the type and number of risk factors (ie no risk factors, hypertension, hyperlipidemia, diabetes mellitus, two risk factors, three risk factors, and prior coronary artery disease).

### 2.5 Outcome adjudication

At least 100 cases with each of the cardiovascular outcomes were assessed by chart review to evaluate the accuracy of ICD‐9 coding. The chart review process included charts from throughout the study's 13‐year follow‐up period. An initial pilot phase was completed and the medical abstractors received feedback from the investigators, resulting in optimization of the coding algorithm. In addition, the sensitivity of the coding algorithm was assessed by reviewing 250 charts which had no coding evidence of any cardiovascular outcomes.

### 2.6 Statistical analysis

The incidence of each cardiovascular endpoint was measured as the rate per 1000 person‐years occurring at any time after the cancer diagnosis. Using the score interval technique,^11^ 95% confidence limits were calculated for each rate. The rates of all‐cause and cancer‐related mortality were also calculated for the larger solid tumor cohort. Next, incidence rates were calculated for each specific solid and liquid tumor of interest. Patients experiencing one endpoint were not excluded from the analysis of the other endpoints such that any individual could be counted as having more than one endpoint.

## 3 RESULTS

### 3.1 Overview of cohort

**Figure 2:**
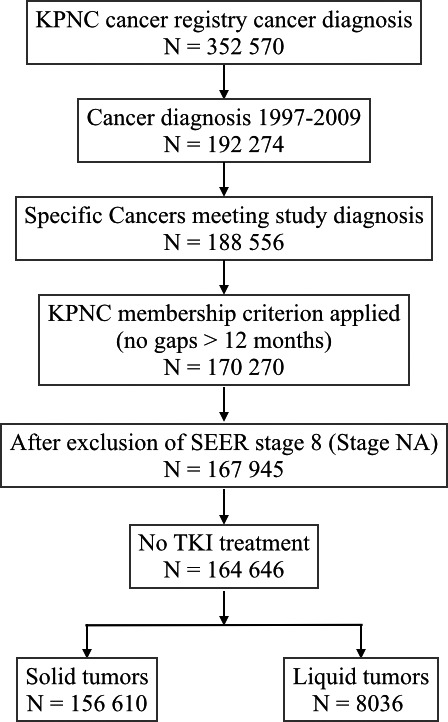
Flow chart describing the sample sizes as the different study inclusion and exclusion criteria were applied to patients with cancer diagnoses between 1997 and 2009

The study population included 156 610 with solid tumors and 8036 with liquid tumors. Figure 2 illustrates the impact of the inclusion and exclusion criteria. The distribution of specific cancer types is shown in Table 1.

**Table 1:** Distribution of cancer types in the study population

Cancer type	Number
All solid tumor cancers	156 610
Renal cancer	3418
Renal cell carcinoma subgroup	2704
Colorectal cancer	13 927
Lung cancer	
Small cell	1769
Non‐small cell	8062
Breast cancer	29 886
Prostate cancer	26 857
Gastrointestinal stromal tumor	36
Hepatocellular cancer	1417
Pancreatic neuroendocrine tumor	103
All liquid tumor cancers	8036
Non‐Hodgkin's Lymphoma ‐ Nodal	4037
Non‐Hodgkin's Lymphoma ‐ Extra nodal	2281
Acute myeloid leukemia	1101
Chronic myeloid leukemia	197
Acute lymphoblastic leukemia	421

The demographic and clinical characteristics of the study's solid and liquid tumor cohorts are shown in Tables 2 and 3, respectively. The study population had a broad age distribution with a mean age of 62. The study cohort was 54% female and included significant African‐American, Asian, and Latino populations. The cohort included a broad range of cancer severity and over 15% of the patients with solid tumors had distant metastases at the time of diagnosis. The patients received multidisciplinary cancer treatments including surgery, chemotherapy, radiation therapy, and/or immunotherapy.

**Table 2:** Demographic and clinical characteristics for patients with solid tumors

Variable	Total 156 610		Men 71 648		Women 84 962	
	N	%	N	%	N	%
Age distribution						
<18 y	725	0.5	299	0.4	426	0.5
18‐39	13 029	8.3	1852	2.6	11 177	13.2
40‐49	15 644	10.0	4174	5.8	11 470	13.5
50‐59	31 072	19.8	13 740	19.2	17 332	20.4
60‐69	41 224	26.3	22 804	31.8	18 420	21.7
70+	54 915	35.1	28 779	40.2	26 136	30.8
Age, mean (SD)	62.2 (15.4)	—	65.7 (12.5)	—	59.3 (16.9)	—
Follow‐up time (y), mean (SD)	4.3 (3.7)	—	4.1 (3.6)	—	4.5 (3.8)	—
Race/ethnicity						
African American	11 992	7.7	5723	8.0	6269	7.4
Asian	14 957	9.6	5907	8.2	9050	10.7
Latino	12 253	7.8	5517	7.7	6736	7.9
Non‐latino white	116 391	74.3	54 119	75.5	62 272	73.3
Other or unknown	1017	0.7	382	0.5	635	0.8
Body mass index >25	67 136	42.9	31 423	43.9	35 713	42.0
Diabetes mellitus	22 201	14.2	12 322	17.2	9879	11.6
Dyslipidemia	52 086	33.3	28 540	39.8	23 546	27.7
Hypertension	77 730	49.6	39 660	55.4	38 070	44.8
Smoking	54 575	34.9	29 880	41.7	24 695	29.1
Coronary artery disease	19 003	12.1	12 702	17.7	6301	7.4
Acute coronary syndrome	8689	5.6	5690	7.9	2999	3.5
Heart failure	11 062	7.1	6161	8.6	4901	5.8
Atrial fibrillation/flutter	10 937	7.0	6526	9.1	4411	5.2
Ischemic stroke	5833	3.7	3159	4.4	2674	3.2
Hemorrhagic stroke	813	0.5	470	0.7	343	0.4
Cardiac arrest	1928	1.2	1288	1.8	640	0.8
Deep venous thrombosis	839	0.5	410	0.6	429	0.5
Pulmonary embolism	1140	0.7	534	0.8	606	0.7
SEER Stage						
0: In situ	25 824	16.5	5717	8.0	20 107	23.7
1: Localized	69 626	44.5	37 073	51.7	32 553	38.3
2: Regional by direct extension	10 061	6.4	5796	8.1	4265	5.0
3: Regional lymph nodes involved	11 546	7.4	2797	3.9	8749	10.3
4: Regional by direct extension & lymph nodes	5884	3.8	2861	4.0	3023	3.6
5: Regional, not specified	435	0.3	231	0.3	204	0.2
7: Distant site(s)/node(s)	25 427	16.2	12 915	18.0	12 512	14.7
9: Unknown	7807	5.0	4258	5.9	3549	4.2
Cancer treatments						
Chemotherapy	37 778	24.1	13 376	18.7	24 402	28.7
Immunotherapy	1890	1.2	1291	1.8	599	0.7
Radiation therapy	40 666	26.0	19 775	27.6	20 891	24.6
Surgical resection	106 538	68.0	36 930	51.5	69 608	81.9

**Table 3:** Demographic and clinical characteristics for patients with liquid tumors

Variable	Total 8036		Men 4352		Women 3684	
	N	%	N	%	N	%
Age distribution						
<18	401	5.0	246	5.7	155	4.2
18‐39	528	6.6	299	6.9	229	6.2
40‐49	794	9.9	462	10.6	332	9.0
50‐59	1433	17.8	785	18.0	648	17.6
60‐69	1754	21.8	979	22.5	775	21.0
70+	3126	38.9	1581	36.3	1545	41.9
Age, mean (SD)	61.2 (19.3)	—	60.1 (19.6)	—	62.5 (18.9)	—
Follow‐up time (y), mean (SD)	3.8 (3.6)	—	3.7 (3.5)	—	4.0 (3.6)	—
Race/ethnicity						
African American	474	5.9	242	5.6	232	6.3
Asian	851	10.6	464	10.7	387	10.5
Latino	751	9.4	419	9.6	332	9.0
Non‐latino White	5923	73.7	3208	73.7	2715	73.7
Other or unknown	37	0.5	19	0.4	18	0.5
Body mass index >25	3170	39.5	1632	37.5	1538	41.8
Diabetes Mellitus	1182	14.7	679	15.6	503	13.7
Dyslipidemia	2487	31.0	1397	32.1	1090	29.6
Hypertension	3932	48.9	2055	47.2	1877	51.0
Smoking	2604	32.4	1617	37.2	987	26.8
Coronary artery disease	1128	14.0	775	17.8	353	9.6
Acute coronary syndrome	535	6.7	341	7.8	194	5.3
Heart failure	726	9.0	421	9.7	305	8.3
Atrial fibrillation/flutter	702	8.7	446	10.3	256	7.0
Ischemic stroke	309	3.9	166	3.8	143	3.9
Hemorrhagic stroke	59	0.7	39	0.9	20	0.5
Cardiac arrest	153	1.9	99	2.3	54	1.5
Deep venous thrombosis	53	0.7	24	0.6	29	0.8
Pulmonary embolism	61	0.8	21	0.5	40	1.1
SEER Stage						
1: Localized	1901	23.7	1009	23.2	892	24.2
2: Regional by direct extension	80	1.0	38	0.9	42	1.1
3: Regional lymph nodes involved	12	0.2	6	0.1	6	0.2
4: Regional by direct extension & lymph nodes	8	0.1	5	0.1	3	0.1
5: Regional, not specified	988	12.3	507	11.7	481	13.1
7: Distant site(s)/node(s)	4767	59.3	2635	60.6	2132	57.9
9: Unknown	280	3.5	152	3.5	128	3.5
Cancer treatments						
Chemotherapy	5281	65.7	2944	67.7	2337	63.4
Immunotherapy	652	8.1	350	8.0	302	8.2
Radiation therapy	1397	17.4	704	16.2	693	18.8
Surgical resection	3667	45.6	1975	45.4	1692	45.9

### 3.2 Cardiovascular endpoints stratified by cancer type

The incidence rates of cardiovascular endpoints according to individual cancer types of interest are shown in Table 4. ACS was most common in patients with small cell or non‐small cell lung cancer. Heart failure and hypertension occurred at a high rate across the spectrum of cancer types. Deep venous thrombosis and pulmonary embolism endpoints were particularly common in patients with lung cancer.

**Table 4:** Incidence of cardiovascular outcomes according to cancer type

Outcome	N				Person‐time (y)	Incidence rate (95% CI)^a^	N	Person‐time (y)	Incidence Rate (95% CI)^a^
	Renal cancer (all types)						Renal cell carcinoma		
Acute coronary syndrome	256				12 383.4	20.7 (18.3‐23.3)	201	10 098.9	19.9 (17.4‐22.8)
Heart failure	354				12 177.6	29.1 (26.2‐32.2)	280	9921.6	28.2 (25.1‐31.7)
Ischemic stroke	122				12 746.0	9.6 (8.0‐11.4)	95	10 390.2	9.14 (7.5‐11.2)
Hemorrhagic stroke	52				12 986.9	4.0 (3.1‐5.3)	46	10 580.4	4.35 (3.3‐5.8)
Cardiac arrest	132				12 910.1	10.2 (8.6‐12.1)	103	10 524.8	9.79 (8.1‐11.9)
Hypertension	347				11 586.3	30.0 (27.0‐33.2)	270	9451.1	28.6 (25.4‐32.1)
Deep venous thrombosis	59				12 968.5	4.55 (3.5‐5.9)	42	10 579.7	3.97 (2.9‐5.4)
Pulmonary embolism	92				12 865.3	7.15 (5.8‐8.8)	77	10 490.0	7.34 (5.9‐9.2)
Cardiovascular death	172				14 460.1	11.9 (10.3‐13.8)	135	11 817.0	11.42 (9.7‐13.5)
	**Colorectal cancer**						**Small cell lung cancer**		
Acute coronary syndrome	914				55 258.6	16.5 (15.5‐17.6)	82	1705.8	48.1 (38.9‐59.3)
Heart failure	1372				54 256.4	25.3 (24.0‐26.6)	122	1643.1	74.3 (62.5‐87.9)
Ischemic stroke	543				56 486.7	9.6 (8.8‐10.5)	40	1720.7	23.3 (17.1‐31.5)
Hemorrhagic stroke	150				57 550.9	2.6 (2.2‐3.1)	22	1743.0	12.6 (8.4‐19.0)
Cardiac arrest	561				57 081.0	9.8 (9.1‐10.7)	86	1729.3	49.7 (40.5‐61.0)
Hypertension	1901				49 098.6	38.7 (37.1‐40.5)	80	1645.2	48.6 (39.2‐60.1)
Deep venous thrombosis	348				57 081.3	6.1 (5.5‐6.8)	22	1734.7	12.7 (8.4‐19.1)
Pulmonary embolism	369				57 074.2	6.5 (5.8‐7.2)	46	1712.1	26.9 (20.2‐35.7)
Cardiovascular death	810				63 546.3	12.8 (11.9‐13.7)	38	1888.6	20.1 (14.7‐27.5)
	**Non‐small cell lung cancer**						**Breast cancer**		
Acute coronary syndrome	501				14 525.4	34.5 (31.6‐37.6)	1089	166 799.3	6.5 (6.2‐6.9)
Heart failure	738				14 151.6	52.2 (48.6‐55.9)	2239	163 420.7	13.7 (13.2‐14.3)
Ischemic stroke	263				14 890.4	17.7 (15.7‐19.9)	877	167 609.2	5.2 (4.9‐5.6)
Hemorrhagic stroke	93				15 085.3	6.2 (5.0‐7.6)	262	169 485.9	1.6 (1.4‐1.8)
Cardiac arrest	357				15 003.9	23.8 (21.5‐26.4)	540	169 137.8	3.2 (2.9‐3.5)
Hypertension	589				13 590.7	43.3 (40.0‐46.9)	5187	144 629.4	35.9 (34.9‐36.8)
Deep venous thrombosis	197				14 991.8	13.1 (11.4‐15.1)	485	168 915.3	2.9 (2.6‐3.1)
Pulmonary embolism	401				14 862.3	27.0 (24.5‐29.7)	467	168 774.5	2.8 (2.5‐3.0)
Cardiovascular death	248				16 286.6	15.2 (13.5‐17.2)	926	187 480.1	4.9 (4.6‐5.3)
	**Prostate cancer**						**Gastrointestinal stromal tumor**		
Acute coronary syndrome	2165				137 258.2	15.8 (15.1‐16.5)	1	194.7	5.1 (0.9‐28.5)
Heart failure	2536				137 066.0	18.5 (17.8‐19.2)	4	194.8	20.5 (8.0‐51.6)
Ischemic stroke	1002				141 479.3	7.1 (6.7‐7.5)	0	196.1	0 (0‐19.2)
Hemorrhagic stroke	350				143 740.3	2.4 (2.2‐2.7)	0	196.1	0 (0‐19.2)
Cardiac arrest	921				142 934.4	6.4 (6.0‐6.9)	3	195.9	15.3 (5.2‐44.1)
Hypertension	3621				127 510.9	28.4 (27.5‐29.3)	9	147.5	61.0 (32.4‐111.9)
Deep venous thrombosis	464				143 382.7	3.2 (3.0‐3.5)	0	196.1	0 (0‐19.2)
Pulmonary embolism	439				143 304.5	3.1 (2.8‐3.4)	2	195.9	10.2 (2.8‐36.5)
Cardiovascular death	1286				160 053.1	8.0 (7.6‐8.5)	0	218.9	0 (0‐17.3)
	**Hepatocellular carcinoma**						**Pancreatic neuroendocrine tumor**		
Acute coronary syndrome	32			1650.4		19.4 (13.8‐27.2)	4	255.6	15.7 (6.1‐39.6)
Heart failure	78			1603.5		48.7 (39.2‐60.3)	9	260.1	34.6 (18.3‐64.4)
Ischemic stroke	11			1666.2		6.6 (3.7‐11.8)	1	275.4	3.6 (0.6‐20.3)
Hemorrhagic stroke	19			1656.9		11.5 (7.4‐17.8)	1	275.6	3.6 (0.6‐20.3)
Cardiac arrest	42			1655.7		25.4 (18.8‐34.1)	6	261.0	23.0 (10.6‐49.2)
Hypertension	60			1555.0		38.6 (30.1‐49.4)	7	260.8	26.8 (13.1‐54.4)
Deep venous thrombosis	9			1659.1		5.4 (2.9‐10.3)	1	274.9	3.6 (0.6‐20.3)
Pulmonary embolism	14			1664.0		8.4 (5.0‐14.1)	1	273.7	3.7 (0.7‐20.4)
Cardiovascular death	22			1843.5		11.9 (7.9‐18.0)	2	294.4	6.8 (1.9‐24.4)
	**Non‐Hodgkin's lymphoma ‐ nodal**						**Non‐Hodgkin's lymphoma ‐ extranodal**		
Acute coronary syndrome	242		15 349.2			15.8 (13.9‐17.9)	122	10 219.7	11.9 (10.0‐14.2)
Heart failure	531		14 735.8			36.0 (33.1‐39.2)	257	9868.7	26.0 (23.1‐29.4)
Ischemic stroke	140		15 565.9			9.0 (7.6‐10.6)	90	10 343.4	8.7 (7.1‐10.7)
Hemorrhagic stroke	49		15 793.1			3.1 (2.4‐4.1)	42	10 461.6	4.0 (3.0‐5.4)
Cardiac arrest	168		15 661.1			10.7 (9.2‐12.5)	72	10 463.0	6.9 (5.5‐8.7)
Hypertension	449		14 161.8			31.7 (28.9‐34.7)	304	9054.5	33.6 (30.1‐37.5)
Deep venous thrombosis	165		15 501.5			10.6 (9.2‐12.4)	50	10 402.0	4.8 (3.7‐6.3)
Pulmonary embolism	121		15 657.1			7.7 (6.5‐9.2)	69	10 354.5	6.7 (5.3‐8.4)
Cardiovascular death	6		15 863.9			0.4 (0.2‐0.8)	7	16 473.2	0.4 (0.2‐0.9)
		**Acute myeloid leukemia**					**Chronic myeloid leukemia**		
Acute coronary syndrome		42			1687.7	24.9 (18.5‐33.5)	13	445.9	29.2 (17.1‐49.2)
Heart failure		124			1574.9	78.7 (66.4‐93.1)	23	440.3	52.2 (35.1‐77.2)
Ischemic stroke		17			1702.4	10.0 (6.2‐15.9)	4	456.7	8.8 (3.4‐22.3)
Hemorrhagic stroke		51			1707.5	29.9 (22.8‐39.1)	13	452.5	28.7 (16.9‐48.5)
Cardiac arrest		45			1719.2	26.2 (19.6‐34.8)	11	451.0	24.4 (13.7‐43.2)
Hypertension		76			1501.0	50.6 (40.6‐62.9)	18	425.9	42.3 (26.9‐65.8)
Deep venous thrombosis		17			1705.6	10.0 (6.2‐15.9)	9	451.2	20.0 (10.5‐37.5)
Pulmonary embolism		14			1720.6	8.1 (4.9‐13.6)	3	456.4	6.6 (2.2‐19.2)
Cardiovascular death		0			7745.0	0 (0‐0.5)	0	1540.0	0 (0‐2.5)
		**Acute lymphoblastic leukemia**							
Acute coronary syndrome		5			1931.9	2.6 (1.1‐6.1)	—	—	—
Heart failure		18			1917.6	9.4 (6.0‐14.8)	—	—	—
Ischemic stroke		8			1930.6	4.1 (2.1‐8.2)	—	—	—
Hemorrhagic stroke		14			1916.8	7.3 (4.4‐12.2)	—	—	—
Cardiac arrest		9			1932.8	4.7 (2.5‐8.8)	—	—	—
Hypertension		52			1741.4	29.9 (22.8‐39.0)	—	—	—
Deep venous thrombosis		5			1931.4	2.6 (1.1‐6.1)	—	—	—
Pulmonary embolism		6			1926.9	3.1 (1.4‐6.8)	—	—	—
Cardiovascular death		0			3010.3	0 (0‐1.3)	—	—	—

Note

An individual patient may experience multiple outcomes. The occurrence of a specific type of outcome censors that patient from experiencing the same outcome again but not other types of outcomes.

a Per 1000 person‐years.

### 3.3 Cause of death

The all‐cause mortality rate among the patients with solid tumors was 74.7 deaths per 1000 person‐years (Table 5). The majority of these deaths were attributed to cancer (54.2 deaths per 1000 person‐years) and a minority were coded as CV deaths (7.6 per 1000 person‐years).

**Table 5:** All‐cause, cancer‐related, and cardiovascular death among patients with solid tumors

	N	Person‐time (years)	Incidence Rate (95% CI)^a^	Days from Cancer Diagnosis to Death^b^ (mean ± SD, median)
All‐cause mortality	57 846	774 671.0	74.7 (74.1‐75.3)	832 ± 1008, 401
Cardiovascular death	5888	774 671.4	7.6 (7.4‐7.8)	1398 ± 1187, 112
Cancer‐related death	41 949	774 671.4	54.2 (53.7‐54.7)	614 ± 813, 285

a Per 1000 person‐years.

b Or censoring.

### 3.4 Cardiovascular endpoints according to risk factor profile

The effects of different cardiovascular risk factors on the incidence rates of the various study outcomes are shown in Table 6. Hypertension, hyperlipidemia, diabetes mellitus, and a history of established coronary artery disease (CAD) were associated with endpoints related to atherosclerosis: ACS, heart failure, ischemic stroke, and cardiovascular death. The effects of these risk factors on the rates of DVT, pulmonary embolism, and hemorrhagic stroke were less marked. Table 6 also demonstrates increasing incidence rates for some outcomes as the number of cardiovascular risk factors increases. For example, the rate of ACS was 12.4 cases per 1000 person‐years in the absence of any risk factors, 22.3 with two risk factors, 34.1 with three risk factors, and 50.4 with a prior history of CAD. There were much smaller differences observed for the venous thromboembolic outcomes, for which atherosclerosis is not a causative mechanism. The incidence of new‐onset hypertension did not increase with the number of cardiovascular risk factors as previous hypertension was an exclusion criterion for this endpoint.

**Table 6:** Cardiovascular outcome rates according to the type and number of risk factors and a history of coronary artery disease

	Incidence rates (95% CI)^a^			
	No risk factors	Hypertension	Hyperlipidemia	Diabetes mellitus
Acute coronary syndrome	12.4 (12.1‐12.7)	20.2 (19.7‐20.7)	21.7 (21.0‐22.4)	29.5 (28.3‐30.7)
Heart failure	18.9 (18.5‐19.2)	32.0 (31.4‐32.7)	29.0 (28.3‐29.8)	43.7 (42.2‐45.3)
Ischemic stroke	7.0 (6.8‐7.2)	10.9 (10.5‐11.3)	9.0 (8.5‐9.4)	13.8 (13.0‐14.6)
Hemorrhagic stroke	2.6 (2.4‐2.7)	3.7 (3.5‐4.0)	3.6 (3.4‐3.9)	4.3 (3.8‐4.8)
Cardiac arrest	6.8 (6.6‐7.0)	10.6 (10.2‐11.0)	10.2 (9.8‐10.7)	14.4 (13.5‐15.2)
Hypertension	32.7 (32.3‐33.2)	12.6 (12.2‐13.0)	22.1 (21.4‐22.8)	20.5 (19.5‐21.6)
Deep venous thrombosis	4.6 (4.5‐4.8)	6.3 (6.0‐6.6)	6.8 (6.4‐7.2)	6.8 (6.2‐7.4)
Pulmonary embolism	5.2 (5.0‐5.4)	7.1 (6.8‐7.5)	7.4 (7.0‐7.8)	6.9 (6.3‐7.5)
All‐cause mortality	74.7 (74.1‐75.3)	109.8 (108.7‐110.9)	100.3 (99.0‐101.6)	133.5 (131.2‐135.8)
Cardiovascular death	7.6 (7.4‐7.8)	13.6 (13.2‐14.0)	11.6 (11.2‐12.1)	17.1 (16.2‐18.0)

	2 Cardiac Risk Factors^b^	3 Cardiac Risk Factors^c^	Coronary Artery Disease	
Acute Coronary Syndrome	22.3 (21.4‐23.2)	34.1 (32.3‐36.0)	50.4 (48.5‐52.3)	—
Heart failure	32.8 (31.7‐33.8)	47.7 (45.6‐50.0)	65.2 (63.1‐67.4)	—
Ischemic stroke	10.6 (10.0‐11.2)	13.3 (12.2‐14.5)	16.2 (15.2‐17.2)	—
Hemorrhagic stroke	3.8 (3.4‐4.1)	5.2 (4.5‐6.0)	5.5 (5.0‐6.2)	—
Cardiac arrest	11.2 (10.6‐11.8)	15.8 (14.6‐17.1)	22.5 (21.3‐23.8)	—
Hypertension	13.2 (12.5‐13.8)	5.2 (4.2‐5.9)	12.2 (11.3‐13.1)	—
Deep venous thrombosis	6.5 (6.0‐6.9)	8.5 (7.7‐9.5)	6.4 (5.8‐7.0)	—
Pulmonary embolism	7.4 (7.0‐7.9)	8.3 (7.5‐9.3)	8.4 (7.7‐9.2)	—
All‐cause mortality	109.4 (107.7‐111.1)	142.6 (139.3‐145.9)	177.9 (174.9‐180.9)	—
Cardiovascular death	13.7 (13.0‐14.3)	18.2 (17.0‐19.5)	32.6 (31.3‐34.1)	—

a Per 1000 person‐years.

b 2/3 of hypertension, hyperlipidemia, and diabetes mellitus.

c Hypertension, hyperlipidemia, and diabetes mellitus.

### 3.5 Endpoint adjudication results

A total of 1052 charts were reviewed, including at least 100 cases for each cardiovascular endpoint. The confirmed endpoint diagnosis rates (true positive rates) ranged from 76% to 91%. The true positive rates for the coding algorithm were stable over time. For the 250‐patient sample without coded outcomes, chart review confirmed the absence of an outcome in 98% of cases.

## 4 DISCUSSION

This analysis offers a comprehensive description of the incidence of cardiovascular outcomes among patients with multiple cancer types. Hypertension and heart failure occurred in high rates among patients with all the cancer types whereas ACS and thromboembolic events occurred most frequently in lung cancer patients. The risk of hemorrhagic stroke was especially high in patients with liquid tumors such as acute and chronic myeloid leukemia. Mortality was common, but a minority of deaths occurred due to cardiovascular causes. Finally, the incidence rates of cardiovascular outcomes were higher among patients with increasing numbers of cardiovascular risk factors.

### 4.1 Comparison with current knowledge

This study showed that new‐onset hypertension was common among patients with cancer; this is not surprising since hypertension has been reported to occur at rates of 10%‐40% among cancer patients, depending on the type and dose of treatment.^12^, ^13^ Variations in reported hypertension rates may be related to the pathophysiology of different cancer types, variable detection and documentation of previously undiagnosed disease during periods of intensive medical observation, specific treatments used for each cancer, differing rates of comorbidities, and varying study follow‐up times.

Prior studies suggest that there is an increased risk of vascular disease such as stroke and ACS among cancer patients.^14^, ^15^, ^16^, ^17^, ^18^ In this study, this was particularly evident in lung cancer patients, who had a particularly high incidence of stroke, consistent with prior studies.^15^ In comparison, an older (1996‐1999) KPNC cohort study of patients with COPD reported an incident stroke rate of approximately 8 per 1000 person‐years versus 17 (non‐small cell) to 23 (small cell) per 1000 person‐years among patients with lung cancer in this study.^19^ A prior KPNC study of population trends in myocardial infarction rates reported an incidence of 2.9 per 1000 person‐years in 1999 which decreased to 2.1 by 2008. Those rates are low compared to what was observed in the lung cancer population in this study: 34.5 ACS (unstable angina plus MI) cases per 1000 person‐years among patients with non‐small cell cancer and 48.1 among those with small‐cell cancer.^20^ Tumor embolism, cerebral metastases, cerebral infections, coagulation disorders, and therapeutic‐side effects all may contribute to cerebrovascular events and ACS in cancer patients.^16^, ^17^

In this study, heart failure endpoints were common, regardless of cancer type, ranging from 9.4 to 78.7 cases per 1000 person‐years. In comparison, a prior KPNC cohort study of patients with diabetes mellitus reported a HF incidence of 4.5 to 9.2 per 1000 person‐years.^21^ In some of the cancers included in this analysis, such as breast cancer and non‐Hodgkin's lymphoma, cardiotoxic medications such as anthracyclines, daunorubicin and doxorubicin, are first line treatments.^5^ In a prior study of 700 breast cancer patients taking anthracyclines, 52 cases of treatment‐induced cardiomyopathy occurred.^22^ Other studies reported a 5‐year cumulative risk of cardiac events including systolic dysfunction and clinical heart failure of 19%‐20% in non‐Hodgkin's lymphoma patients.^23^, ^24^ The cardiotoxicity of anthracyclines partly occurs due to the production of free radicals and reactive oxygen species (ROS) in response to tumor injury resulting in mitochondrial DNA and myocyte damage.^23^, ^24^

### 4.2 Causes of mortality in the study cohort with solid tumors

The incidence rate of cardiovascular mortality was 7.60 per 1000 PY vs. 54.15 per 1000 PY for cancer‐related mortality. Notably, the mean number of days from cancer diagnosis to death was higher in those experiencing cardiovascular mortality compared to cancer‐related mortality (1398 vs 614 respectively). Due to competing risks, it is reasonable to presume that patients with very aggressive cancers experience cancer‐related mortality sooner than cardiovascular‐related mortality and thus a substantial number of patients with the potential to develop cardiovascular disease were censored from the analysis. However, the number of cancer survivors is expected to increase by approximately 70% until the year 2040^25^ and as survival time increases, the number of patients succumbing to cardiovascular disease is also likely to increase.^4^ One prior study reported late cardiotoxicity in 30% of patients 13 years after initiation of cancer treatment.^25^

### 4.3 Risk factors for cardiovascular outcomes

This study shows a trend towards an increased incidence of cardiovascular outcomes among cancer patients with one or more cardiovascular risk factors, a novel finding compared with prior studies which focused on individual risk factors.^22^, ^26^ A history of CAD was associated with the highest incidence rates of the cardiovascular outcomes of interest.

### 4.4 Strengths

The strengths of this study include a large sample size, a long follow‐up period, a robust endpoint adjudication process, and generalizability to the local population. Another strength is the use of data from a cancer registry which provides unbiased population data regarding cancer treatments and survival.

### 4.5 Limitations

This was a retrospective cohort study with intrinsic limitations including missing data, censorship of patients who are lost to follow‐up, and difficulties in comparing outcome timing and variables such as cancer treatment timing. The study was conducted using a cohort of patients selected from 1997 to 2009. However, this cohort was intentionally chosen to quantify the rates of cardiovascular outcomes among cancer patients before the use of newer therapeutic agents. Additionally, it is unclear to what extent KPNC cancer and cardiovascular treatments are generalizable to other health care systems though generalizability may be superior in comparison with studies from academic and/or tertiary care centers.

## 5 CONCLUSIONS

The rates of cardiovascular outcomes among cancer patients included in this analysis were high, regardless of cancer type. Although a majority of deaths during the study period were due to cancer‐related causes, it is important to note that death from cardiovascular causes occurred later after cancer diagnosis, which may be explained by the late effects of therapy‐related cardiotoxicity on cancer survivors. Furthermore, cardiovascular events appeared to occur more commonly in cancer patients with increasing numbers of cardiovascular risk factors and, in particular, a prior history of CAD. Future research is warranted to investigate the role of early cardiovascular screening and the use of cardio‐protective agents in reducing cardiovascular‐related complications in cancer patients.

## CONFLICT OF INTEREST

None of the study authors have conflicts of interest.

## Data Availability

The data that support the findings of this study are available on request from the corresponding author. The data are not publicly available due to privacy or ethical restrictions.
