# Supplementary material for: Incidence rates of cardiovascular outcomes in a community‐based population of cancer patients
Source: Cancer Med. 2019 Oct 30;8(18):7913–23. doi: 10.1002/cam4.2657 (PMC6912043; doi:10.1002/cam4.2657)
Supplement: Supplementary file 1 [file CAM4-8-7913-s001.docx]

**Appendix**

**Table 1. Coding definitions for study variables**

| **Variable** | **Role** | **Data source(s)** | **Operational definition** |
| --- | --- | --- | --- |
| **Cancer type** | Exposure  Stratifying variable | KPNC Cancer Registry  (KPNCCR) | Renal (all types)  Colorectal  Small cell lung  Non-small cell lung  Breast  Prostate  Gastrointestinal stromal tumor  Hepatocellular  Pancreatic neuroendocrine tumor  Brain  Other  * Multiple cancer diagnoses: in the rare cases where more than one kind of cancer diagnosis was present in the registry, the first cancer diagnosis meeting the study criteria was used for the analysis. In the very rare cases where two different cancers were diagnosed on the same date, the PI performed chart review and determined which of the cancer diagnoses was considered clinically more important with regards to treatment decision-making and prognosis. The cancer type for which treatment was based and for which prognosis was made was used for the analysis. |
| **Renal cancer (any type)** | Exposure  Stratifying variable | KPNCCR | Site code C649 |
| **Renal cell carcinoma subgroup** | Exposure  Stratifying variable | KPNCCR | Site code C64.9 and  ICD-O-3 code 8312, 8316-8319, 8005/3, 8050/3, 8310, 8320/3, or 8323/3 |
| **Colorectal cancer** | Exposure  Stratifying variable | KPNCCR | Site codes 21040-21050 |
| **Small cell lung cancer** | Exposure  Stratifying variable | KPNCCR | Site code C33.9, C34.0-34.3, or C34.8-34.9 and  ICD-O-3 code 8041/3, 8042/3, 8043/3, 8044/3, or 8045/3 |
| **Non-small cell lung cancer** | Exposure  Stratifying variable | KPNCCR | Site code C33.9, C34.0-34.3, or C34.8-34.9 and  ICD-O-3 code 814x, 848x, 857x, 807x, 825x, 826x, 831x, 856x, 802.x, 8012/3, 8013/3, 8014/3, 8123/3, or 8011/3 |
| **Breast cancer** | Exposure  Stratifying variable | KPNCCR | Site code 26000 (includes male)^1^ |
| **Prostate cancer** | Exposure  Stratifying variable | KPNCCR | Site code 28010^1^ |
| **Gastrointestinal stromal tumor cancer** | Exposure  Stratifying variable | KPNCCR | Site code C15.0-15.5, C15.8-15.9, C16.0-16.6. C16.8-16.9, C17.0-17.3, C17.8-17.9, C18.0-9, C19.9, C20.9 C21.0-21.2, or C21.8 and  ICD-O-3 code 8936/3 |
| **Hepatocellular cancer** | Exposure  Stratifying variable | KPNCCR | Site code C220  and  ICD-O-3 code 8170 |
| **Pancreatic Neuroendocrine tumor** | Exposure  Stratifying variable | KPNCCR | Site code 250-254, or C257-259  and  ICD-O-3 code 8151/3, 8152/3, 8153/3, 8155/3, 8150/3, 8240/3, 8241/3, or 8246/3 |
| **Non-Hodgkin’s Lymphoma - Nodal** | Exposure  Stratifying variable | KPNCCR | Site code C024, C098,C099, C111,C142, C379,C422, or C770-C779  and  ICD-O-3 code 9590-9596, 9670-9671, 9673, 9675, 9678-9680, 9684, 9688.  9687, 9689-9691, 9695, 9698-9702, 9705, 9708-9709, 9712, 9714-9719, 9724, 9727-9729, 9823, or 9827 |
| **Non-Hodgkin’s Lymphoma -Extranodal** | Exposure  Stratifying variable | KPNCCR | All sites except C024, C098-C099, C111, C142, C379, C422, and C770-C779  and  ICD-O-3 code 9590-9596, 9670-9671, 9673, 9675, 9678-9680, 9684, 9688, 9687, 9689-9691, 9695, 9698-9702, 9705, 9708-9709, 9712, 9714-9719, 9724, or 9727-9729 |
| **Non-Hodgkin’s Lymphoma -Extranodal Subgroup**   - **B cell CLL/small lymphocytic lymphoma** - **Adult T-cell leukemia/lymphoma (HTLV-1+)** | Exposure  Stratifying variable | KPNCCR | All sites except C024, C098-C099, C111, C142, C379, C420-C422, C424, and C770-C779  and  ICD-O-3 codes of 9823 or 9827 |
| **Acute myeloid leukemia** | Exposure  Stratifying variable | KPNCCR | Site code C420, C421, or C424  and  ICD-O-3 codes of 9840/3, 9861/3, 9866/3, 9867/3, 9870/3, 9871/3, 9872/3, 9873/3,  9874/3, 9895/3, 9896/3, 9897/3, 9910/3, 9920/3, 9891/3, or 9911/3 |
| **Chronic myeloid leukemia** | Exposure  Stratifying variable | KPNCCR | Site code C420, C421, or C424  and  ICD-O-3 code 9863/3, 9875/3, 9876/3, 9945/3, or 9946/3 |
| **Acute lymphoblastic leukemia** | Exposure  Stratifying variable | KPNCCR | Site code C420, C421, or C424  and  ICD-O-3 code 9826/3 , 9827/3 , 9828/3, 9835/3, 9836/3, or 9837/3 |
| **TKI/Similar MOA Drug** | Exclusion criterion | KPNC legacy DB2 inpatient and outpatient pharmacy database, KPNC Health Connect Clarity pharmacy database, Computerized Oncology Profile System (COPS), Case Management for Medical Oncology with Laboratory and Outcome Tracking (CAMMOLOT), KP Oncology Management System (BEACON) | Treatment (one or more doses) with any of the following drugs after the cancer diagnosis: Sutent (sunitinib), Nexavar (sorafenib), Avastin (bevacizumab), or Gleevec (imatinib) |
| **Age** | Baseline characteristic | KPNC Clarity/VDW demographics databases | Age on the date of cancer diagnosis |
| **Gender** | Baseline characteristic | KPNC Clarity/VDW demographics databases | Female/ Male |
| **Race/ethnicity** | Baseline characteristic | KPNC Clarity/VDW demographics databases | White, African-American, Asian, Latino, other, or unknown |
| **Body mass index (BMI)** | Baseline characteristic | At the time of the most recent encounter prior to the cancer diagnosis, imported directly from KPNC VDW diagnosis database and VDW vital signs database | Calculated from height and weight data when necessary. |
| **Diabetes Mellitus** | Baseline characteristic and risk factor | KPNC Diabetes Registry | Patient listed in the registry during the time window of 1/1/1996 to the day before the cancer diagnosis. |
| **Dyslipidemia** | Baseline characteristic and risk factor | KPNC VDW diagnosis databases | - ICD-9 codes: 272.0x, 272.1x, 272.2x, 272.4x - Accepted any codes during the time window of 1/1/1996 to the day before the cancer diagnosis. |
| **History of Hypertension** | Baseline characteristic and risk factor | KPNC VDW diagnosis database and KPNC legacy outpatient pharmacy database | - ICD-9 codes: 401.0, 401.1, or 401.9 - Accepted any codes during the time window of 1/1/1996 to the day before the cancer diagnosis. |
| **Smoking** | Baseline characteristic and risk factor | KPNC VDW diagnosis database | - ICD-9 codes 305.1 or V15.82 - Accepted any codes during the time window of 1/1/1996 to the day before the cancer diagnosis. |
| **Year of cancer diagnosis** | Baseline characteristic | KPNCCR | 1997-2009 |
| **Cancer disease severity**  **(SEER staging)** | Baseline characteristic | KPNCCR | 0 In situ  1 Localized only  2 Regional by direct extension only  3 Regional lymph nodes involved only  4 Regional by BOTH direct extension AND lymph node involvement  5 Regional, NOS (Not Otherwise Specified)  7 Distant site(s)/node(s) involved  9 Unknown if extension or metastasis (unstaged, unknown, or unspecified)  Death certificate only case |
| **Chemotherapy** | Clinical characteristic | KPNCCR | - Treatment (inpatient or outpatient) with any chemotherapy agent (other than the TKI/similar MOA types - Timing requirement: treatment dispensed any time after the cancer diagnosis. |
| **Immunotherapy** | Clinical characteristic | KPNCCR | Treatment (inpatient or outpatient) with immunotherapy |
| **Radiation therapy** | Clinical characteristic | KPNCCR | Treatment with radiation therapy |
| **Surgical resection** | Clinical characteristic | KPNCCR | Surgical cancer treatment |
| **Other systemic cancer therapy** | Clinical characteristic | KPNCCR | Received any other type of cancer treatment beyond the 4 types described above. |
| **Acute coronary syndrome** | Outcome | KPNC VDW diagnosis database | ICD9 codes: 411.1 or 410.x  Requires: Hospitalization with an ACS discharge diagnosis code (above) after the cancer diagnosis (allows a prior history of ACS that preceded cancer) ***or*** a new diagnosis code for ACS appearing in the outpatient medical record after the cancer diagnosis. |
| **Heart failure** | Outcome | KPNC VDW diagnosis database | ICD9 codes: 428.x, 402.01, 402.11, or 402.91  Requires: Hospitalization with a HF diagnosis code (above) after the cancer diagnosis ***or*** a diagnosis of HF appearing in the outpatient medical record after the cancer diagnosis.  Timing exclusion: if a HF code (above) was present from any source (inpatient, outpatient, transfer, etc) within one year prior to the cancer diagnosis |
| **Incident Ischemic Stroke** | Outcome | KPNC VDW diagnosis database | ICD9 codes: 433.x1, 434.x1, or 436  Requires: Hospitalization with a stroke diagnosis code (above) after the cancer diagnosis ***or*** a new diagnosis of stroke appearing in the outpatient medical record after the cancer diagnosis.  Timing exclusion: if an ischemic stroke code (above) was present from any source (inpatient, outpatient, transfer, etc) at any time prior to the cancer diagnosis. |
| **Hemorrhagic Stroke** | Outcome | KPNC VDW diagnosis database | ICD9 codes: 430, 431, or 432.x  Requires: Hospitalization with a hemorrhagic stroke diagnosis code (above) after the cancer diagnosis (allows a prior history of hemorrhagic stroke that preceded cancer) ***or*** a new diagnosis code for hemorrhagic stroke appearing in the outpatient medical record after the cancer diagnosis. |
| **Cardiac arrest/sustained ventricular arrhythmia** | Outcome | KPNC VDW diagnosis database | ICD9 codes: 427.1, 427.4, or 427.5  Requires: Hospitalization with a cardiac arrest diagnosis code (above) after the cancer diagnosis (allows a prior history of arrest that preceded cancer) ***or*** a new diagnosis code for cardiac arrest appearing in the outpatient medical record after the cancer diagnosis. |
| **Hypertension (HTN)** | Outcome | KPNC VDW diagnosis database and KPNC legacy outpatient pharmacy database | ICD9 code: 401.0, 401.1, or 401.9  Requires: Hospitalization with a HTN diagnosis code (above) after the cancer diagnosis ***or*** a diagnosis of HTN appearing in the outpatient medical record after the cancer diagnosis.  Exclusions:  Timing: if the HTN code (above) was present from any source (inpatient, outpatient, transfer, etc) within one year prior to the cancer diagnosis.  Pharmacy: if a single outpatient prescription for an antihypertensive medication was filled within 1 year prior to the cancer diagnosis. |
| **Deep venous thrombosis (DVT)** | Outcome | KPNC VDW diagnosis database | ICD9 codes: 451.11, 451.19, 451.2, 451.40, 451.41, 451.42, 451.81, 451.83, 451.89, or 453.4  Requires: Hospitalization with a DVT diagnosis code (above) after the cancer diagnosis (allows a prior history of DVT that preceded cancer) ***or*** a new diagnosis of DVT appearing in the outpatient medical record after the cancer diagnosis. |
| **Pulmonary embolism (PE)** | Outcome | KPNC VDW diagnosis database | ICD9 codes: 415.1x, 415.11, or 415.19  Requires: Hospitalization with a PE diagnosis code (above) after the cancer diagnosis (allows a prior history of PE that preceded cancer) ***or*** a new diagnosis code for PE appearing in the outpatient medical record after the cancer diagnosis. |
| **All-Cause Mortality** | Outcome | KPNC VDW death and VDW cause of death database, KPNC department mortality tape and KPNC cancer registry | Requires: Death from any cause occurring any time after the cancer diagnosis. |
| **Cardiovascular Mortality** | Outcome | KPNC VDW death and VDW cause of death database, KPNC department mortality tape and KPNC cancer registry | Requires: Death occurring any time after the cancer diagnosis (confirmed by KP or state DBs), associated with one of the codes below. The code needs to be listed as the primary cause of death.  ICD codes: any of the codes below will be accepted.  ICD9: Any code meeting criteria for the study’s individual endpoints  ICD10 death codes: I00-I99  ICD9 death codes: 390-459 (these are older codes which should only be present prior to 1996). |

**Table 2: Demographic characteristics and co-morbidities according to type of cancer**

|  | Cancer Type | | | | | | | | | | | |
| --- | --- | --- | --- | --- | --- | --- | --- | --- | --- | --- | --- | --- |
| Characteristic | Renal any type | | Renal cell carcinoma subgroup | | Colorectal | | SCLC | | NSCLC | | Breast | |
|  | N | % | N | % | N | % | N | % | N | % | N | % |
| Total | 3418 | 100 | 2704 | 100 | 13927 | 100 | 1769 | 100 | 8062 | 100 | 29886 | 100 |
| Age  (mean ± sd) | 63.05 | 14.3 | 62.76 | 12.9 | 67.53 | 12.91 | 67.94 | 9.65 | 68.41 | 10.41 | 60.89 | 12.8 |
| <18 | 36 | 1.05 | 1 | 0.04 | 0 | 0 | 0 | 0 | 1 | 0.01 | 1 |  |
| 18-39 | 118 | 3.45 | 103 | 3.81 | 246 | 1.77 | 7 | 0.4 | 37 | 0.46 | 1077 | 3.6 |
| 40-49 | 388 | 11.35 | 331 | 12.24 | 970 | 6.96 | 60 | 3.39 | 323 | 4.01 | 5057 | 16.92 |
| 50-59 | 754 | 22.06 | 646 | 23.89 | 2660 | 19.1 | 288 | 16.28 | 1227 | 15.22 | 8000 | 26.77 |
| 60-69 | 903 | 26.42 | 729 | 26.96 | 3388 | 24.33 | 580 | 32.79 | 2535 | 31.44 | 7729 | 25.86 |
| 70+ | 1219 | 35.66 | 894 | 33.06 | 6662 | 47.84 | 834 | 47.15 | 3939 | 48.86 | 8022 | 26.84 |
| Race/Ethnicity |  |  |  |  |  |  |  |  |  |  |  |  |
| African American | 297 | 8.69 | 218 | 8.06 | 1148 | 8.24 | 101 | 5.71 | 741 | 9.19 | 2303 | 7.71 |
| Asian | 268 | 7.84 | 213 | 7.88 | 1600 | 11.49 | 72 | 4.07 | 792 | 9.82 | 3738 | 12.51 |
| Latino | 421 | 12.32 | 366 | 13.54 | 1121 | 8.05 | 91 | 5.14 | 375 | 4.65 | 2164 | 7.24 |
| Non-Latino White | 2423 | 70.89 | 1903 | 70.38 | 10003 | 71.82 | 1500 | 84.79 | 6132 | 76.06 | 21590 | 72.24 |
| Other  or unknown | 9 | 0.26 | 4 | 0.15 | 55 | 0.39 | 5 | 0.28 | 22 | 0.27 | 91 | 0.3 |
| Body mass index >25 | 1868 | 54.65 | 1491 | 55.14 | 6097 | 43.78 | 651 | 36.8 | 2894 | 35.9 | 12849 | 42.99 |
| Diabetes Mellitus | 732 | 21.42 | 612 | 22.63 | 2596 | 18.64 | 317 | 17.92 | 1219 | 15.12 | 3332 | 11.15 |
| Dyslipidemia | 1442 | 42.19 | 1134 | 41.94 | 5181 | 37.2 | 714 | 40.36 | 3099 | 38.44 | 8600 | 28.78 |
| Hypertension | 2307 | 67.5 | 1823 | 67.42 | 7787 | 55.91 | 1068 | 60.37 | 4690 | 58.17 | 13531 | 45.28 |
| Smoking | 1467 | 42.92 | 1112 | 41.12 | 4661 | 33.47 | 1394 | 78.8 | 5229 | 64.86 | 6551 | 21.92 |
| Coronary artery disease | 561 | 16.41 | 440 | 16.27 | 2115 | 15.19 | 372 | 21.03 | 1472 | 18.26 | 1768 | 5.92 |
| Acute Coronary Syndrome | 270 | 7.9 | 215 | 7.95 | 1011 | 7.26 | 177 | 10.01 | 651 | 8.07 | 825 | 2.76 |
| Heart Failure | 364 | 10.65 | 290 | 10.72 | 1412 | 10.14 | 229 | 12.95 | 908 | 11.26 | 1218 | 4.08 |
| Atrial fibrillation/flutter | 338 | 9.89 | 246 | 9.1 | 1379 | 9.9 | 190 | 10.74 | 876 | 10.87 | 1185 | 3.97 |
| Ischemic Stroke | 185 | 5.41 | 147 | 5.44 | 666 | 4.78 | 112 | 6.33 | 429 | 5.32 | 704 | 2.36 |
| Hemorrhagic Stroke | 19 | 0.56 | 15 | 0.55 | 76 | 0.55 | 8 | 0.45 | 47 | 0.58 | 92 | 0.31 |
| Cardiac Arrest | 70 | 2.05 | 53 | 1.96 | 254 | 1.82 | 42 | 2.37 | 158 | 1.96 | 145 | 0.49 |
| Deep venous thrombosis | 21 | 0.61 | 16 | 0.59 | 95 | 0.68 | 9 | 0.51 | 57 | 0.71 | 88 | 0.29 |
| Pulmonary embolism | 41 | 1.2 | 34 | 1.26 | 126 | 0.9 | 19 | 1.07 | 100 | 1.24 | 109 | 0.36 |
| SEER Stage |  |  |  |  |  |  |  |  |  |  |  |  |
| 0 | 66 | 1.93 | 0 | 0 | 537 | 3.86 | 0 | 0 | 2 | 0.02 | 5165 | 17.28 |
| 1 | 2168 | 63.43 | 1824 | 67.46 | 5428 | 38.97 | 63 | 3.56 | 1718 | 21.31 | 16243 | 54.35 |
| 2 | 439 | 12.84 | 324 | 11.98 | 2190 | 15.72 | 63 | 3.56 | 622 | 7.72 | 317 | 1.06 |
| 3 | 37 | 1.08 | 25 | 0.92 | 1256 | 9.02 | 162 | 9.16 | 737 | 9.14 | 6449 | 21.58 |
| 4 | 55 | 1.61 | 31 | 1.15 | 1802 | 12.94 | 149 | 8.42 | 662 | 8.21 | 574 | 1.92 |
| 5 | 2 | 0.06 | 2 | 0.07 | 8 | 0.06 | 5 | 0.28 | 29 | 0.36 | 5 | 0.02 |
| 7 | 588 | 17.2 | 462 | 17.09 | 2336 | 16.77 | 1298 | 73.37 | 4149 | 51.46 | 861 | 2.88 |
| 9 | 63 | 1.84 | 36 | 1.33 | 370 | 2.66 | 29 | 1.64 | 143 | 1.77 | 272 | 0.91 |
| Cancer treatments |  |  |  |  |  |  |  |  |  |  |  |  |
| Chemotherapy | 181 | 5.3 | 86 | 3.18 | 5329 | 38.26 | 1318 | 74.51 | 3470 | 43.04 | 11212 | 37.52 |
| Immunotherapy | 87 | 2.55 | 80 | 2.96 | 20 | 0.14 | 4 | 0.23 | 8 | 0.1 | 54 | 0.18 |
| Radiation therapy | 161 | 4.71 | 117 | 4.33 | 1709 | 12.27 | 663 | 37.48 | 2724 | 33.79 | 12492 | 41.8 |
| Surgical resection | 2764 | 80.87 | 2200 | 81.36 | 12224 | 87.77 | 49 | 2.77 | 2551 | 31.64 | 28398 | 95.02 |

|  | Cancer Type | | | | | | | | | | | |
| --- | --- | --- | --- | --- | --- | --- | --- | --- | --- | --- | --- | --- |
| Characteristic | Prostate | | GIST | | Hepatocellular | | Pancreatic Neuroendocrine tumor | | Solid Total | | NHL nodal | |
|  | N | % | N | % | N | % | N | % | N | % | N | % |
| Total | 26857 | 100 | 36 | 100 | 1417 | 100 | 103 | 100 | 156610 | 100 | 4037 | 100 |
| Age  (mean ± sd) | 66.62 | 8.97 | 59.97 | 13.94 | 63.62 | 11.97 | 61.5 | 15.17 | 62.19 | 15.41 | 63.87 | 15.42 |
| <18 | 2 | 0.01 | 0 | 0 | 1 | 0.07 | 1 | 0.97 | 725 | 0.46 | 40 | 0.99 |
| 18-39 | 5 | 0.02 | 3 | 8.33 | 28 | 1.98 | 7 | 6.8 | 13029 | 8.32 | 212 | 5.25 |
| 40-49 | 542 | 2.02 | 7 | 19.44 | 126 | 8.89 | 12 | 11.65 | 15644 | 9.99 | 428 | 10.6 |
| 50-59 | 5385 | 20.05 | 7 | 19.44 | 413 | 29.15 | 22 | 21.36 | 31072 | 19.84 | 781 | 19.35 |
| 60-69 | 11028 | 41.06 | 9 | 25 | 368 | 25.97 | 28 | 27.18 | 41224 | 26.32 | 948 | 23.48 |
| 70+ | 9895 | 36.84 | 10 | 27.78 | 481 | 33.94 | 33 | 32.04 | 54915 | 35.06 | 1628 | 40.33 |
| Race/Ethnicity |  |  |  |  |  |  |  |  |  |  |  |  |
| African American | 3031 | 11.29 | 4 | 11.11 | 129 | 9.1 | 10 | 9.71 | 11992 | 7.66 | 231 | 5.72 |
| Asian | 2050 | 7.63 | 7 | 19.44 | 373 | 26.32 | 15 | 14.56 | 14957 | 9.55 | 355 | 8.79 |
| Latino | 2100 | 7.82 | 2 | 5.56 | 218 | 15.38 | 11 | 10.68 | 12253 | 7.82 | 342 | 8.47 |
| Non-Latino White | 19495 | 72.59 | 23 | 63.89 | 687 | 48.48 | 66 | 64.08 | 116391 | 74.32 | 3095 | 76.67 |
| Other or unknown | 181 | 0.67 | 0 | 0 | 10 | 0.71 | 1 | 0.97 | 1017 | 0.65 | 14 | 0.35 |
| Body mass index >25 | 12457 | 46.38 | 17 | 47.22 | 686 | 48.41 | 60 | 58.25 | 67136 | 42.87 | 1714 | 42.46 |
| Diabetes Mellitus | 4042 | 15.05 | 5 | 13.89 | 484 | 34.16 | 33 | 32.04 | 22201 | 14.18 | 617 | 15.28 |
| Dyslipidemia | 11562 | 43.05 | 11 | 30.56 | 391 | 27.59 | 43 | 41.75 | 52086 | 33.26 | 1353 | 33.51 |
| Hypertension | 15098 | 56.22 | 15 | 41.67 | 853 | 60.2 | 56 | 54.37 | 77730 | 49.63 | 2070 | 51.28 |
| Smoking | 8288 | 30.86 | 11 | 30.56 | 718 | 50.67 | 38 | 36.89 | 54575 | 34.85 | 1408 | 34.88 |
| Coronary artery disease | 3914 | 14.57 | 6 | 16.67 | 191 | 13.48 | 11 | 10.68 | 19003 | 12.13 | 584 | 14.47 |
| Acute Coronary Syndrome | 1711 | 6.37 | 1 | 2.78 | 94 | 6.63 | 5 | 4.85 | 8689 | 5.55 | 268 | 6.64 |
| Heart Failure | 1437 | 5.35 | 3 | 8.33 | 166 | 11.71 | 6 | 5.83 | 11062 | 7.06 | 357 | 8.84 |
| Atrial fibrillation/flutter | 1743 | 6.49 | 4 | 11.11 | 101 | 7.13 | 3 | 2.91 | 10937 | 6.98 | 374 | 9.26 |
| Ischemic Stroke | 823 | 3.06 | 2 | 5.56 | 72 | 5.08 | 3 | 2.91 | 5833 | 3.72 | 147 | 3.64 |
| Hemorrhagic Stroke | 118 | 0.44 | 0 | 0 | 18 | 1.27 | 0 | 0 | 813 | 0.52 | 17 | 0.42 |
| Cardiac Arrest | 351 | 1.31 | 2 | 5.56 | 15 | 1.06 | 3 | 2.91 | 1928 | 1.23 | 78 | 1.93 |
| Deep venous thrombosis | 96 | 0.36 | 0 | 0 | 3 | 0.21 | 0 | 0 | 839 | 0.54 | 20 | 0.5 |
| Pulmonary embolism | 122 | 0.45 | 0 | 0 | 8 | 0.56 | 1 | 0.97 | 1140 | 0.73 | 37 | 0.92 |
| SEER Stage |  |  |  |  |  |  |  |  |  |  |  |  |
| 0 | 4 | 0.01 | 0 | 0 | 0 | 0 | 0 | 0 | 25824 | 16.49 | 0 | 0 |
| 1 | 21780 | 81.1 | 22 | 61.11 | 595 | 41.99 | 25 | 24.27 | 69626 | 44.46 | 619 | 15.33 |
| 2 | 2243 | 8.35 | 4 | 11.11 | 302 | 21.31 | 5 | 4.85 | 10061 | 6.42 | 41 | 1.02 |
| 3 | 132 | 0.49 | 0 | 0 | 31 | 2.19 | 6 | 5.83 | 11546 | 7.37 | 5 | 0.12 |
| 4 | 112 | 0.42 | 2 | 5.56 | 20 | 1.41 | 4 | 3.88 | 5884 | 3.76 | 0 | 0 |
| 5 | 9 | 0.03 | 1 | 2.78 | 25 | 1.76 | 0 | 0 | 435 | 0.28 | 678 | 16.79 |
| 7 | 1294 | 4.82 | 5 | 13.89 | 317 | 22.37 | 60 | 58.25 | 25427 | 16.24 | 2536 | 62.82 |
| 9 | 1283 | 4.78 | 2 | 5.56 | 127 | 8.96 | 3 | 2.91 | 7807 | 4.98 | 158 | 3.91 |
| Cancer treatments |  |  |  |  |  |  |  |  |  |  |  |  |
| Chemotherapy | 111 | 0.41 | 4 | 11.11 | 423 | 29.85 | 37 | 35.92 | 37778 | 24.12 | 2853 | 70.67 |
| Immunotherapy | 12 | 0.04 | 0 | 0 | 4 | 0.28 | 0 | 0 | 1890 | 1.21 | 469 | 11.62 |
| Radiation therapy | 10254 | 38.18 | 1 | 2.78 | 38 | 2.68 | 7 | 6.8 | 40666 | 25.97 | 697 | 17.27 |
| Surgical resection | 7004 | 26.08 | 31 | 86.11 | 213 | 15.03 | 35 | 33.98 | 106538 | 68.03 | 1197 | 29.65 |

|  | Cancer Type | | | | | | | |
| --- | --- | --- | --- | --- | --- | --- | --- | --- |
| Characteristic | NHL extranodal | | AML | | CML | | ALL | |
|  | N | % | N | % | N | % | N | % |
| Total | 2281 | 100 | 1101 | 100 | 197 | 100 | 421 | 100 |
| Age  (mean ± sd) | 63.07 | 16.07 | 62.35 | 19.57 | 67.37 | 17.82 | 20.09 | 22.74 |
| <18 | 24 | 1.05 | 48 | 4.36 | 8 | 4.06 | 281 | 66.75 |
| 18-39 | 166 | 7.28 | 91 | 8.27 | 4 | 2.03 | 55 | 13.06 |
| 40-49 | 233 | 10.21 | 99 | 8.99 | 16 | 8.12 | 18 | 4.28 |
| 50-59 | 440 | 19.29 | 170 | 15.44 | 19 | 9.64 | 23 | 5.46 |
| 60-69 | 529 | 23.19 | 213 | 19.35 | 40 | 20.3 | 24 | 5.7 |
| 70+ | 889 | 38.97 | 480 | 43.6 | 110 | 55.84 | 20 | 4.75 |
| Race/Ethnicity |  |  |  |  |  |  |  |  |
| African American | 122 | 5.35 | 74 | 6.72 | 13 | 6.6 | 34 | 8.08 |
| Asian | 284 | 12.45 | 140 | 12.72 | 11 | 5.58 | 61 | 14.49 |
| Latino | 190 | 8.33 | 94 | 8.54 | 15 | 7.61 | 110 | 26.13 |
| Non-Latino White | 1673 | 73.35 | 786 | 71.39 | 156 | 79.19 | 214 | 50.83 |
| Other or unknown | 12 | 0.53 | 7 | 0.64 | 2 | 1.02 | 2 | 0.48 |
| Body mass index >25 | 851 | 37.31 | 449 | 40.78 | 70 | 35.53 | 86 | 20.43 |
| Diabetes Mellitus | 329 | 14.42 | 173 | 15.71 | 34 | 17.26 | 29 | 6.89 |
| Dyslipidemia | 733 | 32.14 | 316 | 28.7 | 48 | 24.37 | 37 | 8.79 |
| Hypertension | 1133 | 49.67 | 552 | 50.14 | 112 | 56.85 | 66 | 15.68 |
| Smoking | 682 | 29.9 | 411 | 37.33 | 63 | 31.98 | 40 | 9.5 |
| Coronary artery disease | 313 | 13.72 | 188 | 17.08 | 31 | 15.74 | 13 | 3.09 |
| Acute Coronary Syndrome | 142 | 6.23 | 104 | 9.45 | 17 | 8.63 | 5 | 1.19 |
| Heart Failure | 170 | 7.45 | 158 | 14.35 | 32 | 16.24 | 10 | 2.38 |
| Atrial fibrillation/flutter | 166 | 7.28 | 130 | 11.81 | 27 | 13.71 | 6 | 1.43 |
| Ischemic Stroke | 89 | 3.9 | 59 | 5.36 | 11 | 5.58 | 3 | 0.71 |
| Hemorrhagic Stroke | 15 | 0.66 | 22 | 2 | 3 | 1.52 | 2 | 0.48 |
| Cardiac Arrest | 39 | 1.71 | 29 | 2.63 | 4 | 2.03 | 3 | 0.71 |
| Deep venous thrombosis | 14 | 0.61 | 16 | 1.45 | 2 | 1.02 | 1 | 0.24 |
| Pulmonary embolism | 9 | 0.39 | 11 | 1 | 3 | 1.52 | 1 | 0.24 |
| SEER Stage |  |  |  |  |  |  |  |  |
| 0 | 0 | 0 | 0 | 0 | 0 | 0 | 0 | 0 |
| 1 | 1282 | 56.2 | 0 | 0 | 0 | 0 | 0 | 0 |
| 2 | 39 | 1.71 | 0 | 0 | 0 | 0 | 0 | 0 |
| 3 | 7 | 0.31 | 0 | 0 | 0 | 0 | 0 | 0 |
| 4 | 8 | 0.35 | 0 | 0 | 0 | 0 | 0 | 0 |
| 5 | 310 | 13.59 | 0 | 0 | 0 | 0 | 0 | 0 |
| 7 | 512 | 22.45 | 1101 | 100 | 197 | 100 | 421 | 100 |
| 9 | 123 | 5.39 | 0 | 0 | 0 | 0 | 0 | 0 |
| Cancer treatments |  |  |  |  |  |  |  |  |
| Chemotherapy | 1210 | 53.05 | 730 | 66.3 | 92 | 46.7 | 397 | 94.3 |
| Immunotherapy | 143 | 6.27 | 14 | 1.27 | 11 | 5.58 | 15 | 3.56 |
| Radiation therapy | 578 | 25.34 | 75 | 6.81 | 5 | 2.54 | 42 | 9.98 |
| Surgical resection | 752 | 32.97 | 1101 | 100 | 197 | 100 | 421 | 100 |
